# Validity and reliability of the Persian version of the emotional labour scale for nurses

**DOI:** 10.1186/s12912-025-03587-8

**Published:** 2025-07-15

**Authors:** Saeed Barzegari, Elyas Hosseinzadeh Younesi, Kurosh Jodaki, Ibrahim Arpaci, Negar Esmaeelzadeh, Seyed Alireza Hasani

**Affiliations:** 1 Department of Paramedicine, Amol School of Paramedical Sciences, Mazandaran University of Medical Sciences, Sari, Iran; 2 Nursing Research Center, Gonabad University of Medical Sciences, Gonabad, Iran; 3 School of Nursing and Midwifery, Shohadaye Haft-e Tir Hospital, Iran University of Medical Sciences, Tehran, Iran; 4 Department of Computer Science and Engineering, College of Informatics, Korea University, Seoul, 02841 Republic of Korea; 5 Department of Software Engineering, Faculty of Engineering and Natural Sciences, Bandirma Onyedi Eylul University, Balikesir, Turkey; 6 Management Information Systems, College of Business Administration, Gulf University for Science and Technology, Mishref, Kuwait; 7 Student Research Committee, Mazandaran University of Medical Sciences, Amol, Iran; 8 Department of Nursing, Amol Faculty of Nursing and Midwifery, Mazandaran University of Medical Sciences, Sari, Iran

**Keywords:** Emotional labour, Nurses, Scale validation, Reliability, Psychometrics

## Abstract

**Background:**

Emotional labour plays a critical role in nurses’ daily responsibilities and is deeply embedded in the fabric of professional nursing care. This study aims to assess the reliability and validity of the Persian version of the Emotional Labour Scale (ELS) for nurses.

**Methods:**

The study population consisted of nurses working in hospitals in Amol, Iran, and a methodological cross-sectional approach was applied using a convenience sampling method. To assess the ELS, a back-and-forth procedure was utilized to translate it into Persian. Content and face validity were assessed, and exploratory factor analyses (EFA) and confirmatory factor analyses (CFA) were conducted. Reliability was evaluated using McDonald’s omega and Cronbach’s alpha coefficient.

**Results:**

As a result of the EFA, four items were removed, leaving a final selection of 14 items. The items were categorized under three factors: “Emotional Control Effort in the Profession,” “Patient-Focused Emotional Suppression,” and “Emotional Display According to Norms,” which explained 61.70% of the total variance. CFA results revealed that the model provided a good fit for the study data (χ² = 131.788, df = 67, χ²/df = 1.967, CFI = 0.94, IFI = 0.95, TLI = 0.93, GFI = 0.92, PCFI = 0.69, PNFI = 0.66, and RMSEA = 0.067), and the subscales showed high internal consistency with Cronbach’s alpha coefficients ranging from 0.643 to 0.865.

**Conclusions:**

The Persian version of the ELS with 14 items and a three-factor structure showed satisfactory validity and reliability among Iranian nurses. Its strong psychometric properties support its use for assessing emotional labour and informing interventions to promote nurses’ emotional well-being.

## Introduction

Care is a structured process that involves selflessly assisting others and fostering interpersonal relationships, beginning with an understanding of individuals’ challenges [1]. Nurses, as essential pillars of healthcare, are entrusted with patient care and addressing both their patients’ emotional needs and their own [2]. In addition, nurses are expected to demonstrate qualities such as sensitivity, empathy, compassion, and emotional caregiving in their interactions with patients [3]. This responsibility requires the use of emotional expression to nurture and sustain relationships with patients, a concept known as “emotional labour” [4].

In the healthcare field, emotional labour encompasses the skills and behaviors that medical professionals utilize to care for others and acknowledge their emotions [5, 6]. As a fundamental aspect of nursing, emotional labour significantly influences the quality of patient care and the nurses’ psychological well-being [7, 8]. By empathizing with and addressing patients’ emotions, nurses foster a sense of comfort and security, which can potentially alleviate anxiety and stress levels [9]. Positive emotional connections between nurses and patients enhance trust and improve communication, making it easier for patients to articulate their concerns and challenges [10]. Research indicates that patients who receive emotional care tend to recover more quickly and experience shorter hospital stays [11]. Cultivating strong emotional bonds and trust between nurses and patients can also reduce the frequency and urgency of medical interventions, thereby lightening nurses’ workloads and alleviating the strain on healthcare systems [12].

While emotional labour offers significant benefits for patients and the healthcare system, it can also lead to adverse outcomes for nurses. Prolonged emotional strain in the workplace can lead to emotional fatigue, which diminishes the ability to deliver high-quality care and respond effectively to patients’ needs [13]. Excessive emotional labour may also contribute to burnout, manifesting as decreased job performance, increased absenteeism, or even resignation, negatively impacting not only nurses but the entire healthcare system [12, 14, 15]. Furthermore, it can also lead to mental health problems such as depression and anxiety, and somatic issues such as sleep disorders, headaches, and gastrointestinal problems [16, 17]. These challenges can profoundly affect both the personal and professional lives of nurses [18].

Given these potential benefits and drawbacks, it is essential to assess and measure emotional labour among nurses using reliable questionnaires within healthcare systems. Such evaluations assist healthcare administrators in developing strategies to manage emotional pressures and mitigate their adverse effects [19]. Measuring emotional labour also enhances the understanding of emotional demands, promotes effective emotional management, improves the quality of care, deepens insights into patients’ feelings, prevents emotional burnout, safeguards nurses’ mental health, and supports their training and empowerment [14, 20]. These assessments can inform the creation of training programs designed to enhance nurses’ emotional and communication skills, foster a positive workplace culture, and improve clinical outcomes [21].

Several frameworks and instruments have been developed to assess emotional labour. Grandey provided a conceptual model that defines emotional labour as the regulation of emotions to align with organizational display rules, emphasizing the role of internal emotional regulation processes [22]. In contrast, Brotheridge and Lee developed an Emotional Labour Scale that distinguishes between surface acting and deep acting, drawing attention to the psychological strain caused by emotional dissonance [23], while Diefendorff introduced the Emotional Labour Strategies Questionnaire, which focuses on specific emotional regulation strategies and their implications for job performance [24]. None of these scales is specifically designed for nurses. Furthermore, the emotional labour experienced by nurses differs significantly from that of other public service employees due to the unique emotional challenges present in healthcare settings [25]. Nurses frequently manage patients’ depression, anger, anxiety, suffering, and trauma while maintaining positive emotional expressions, core elements of the nursing role [26]. Therefore, questionnaires designed to measure emotional labour in nursing should be tailored to the profession’s unique aspects to ensure effectiveness across various care scenarios.

To address this gap, Hong developed the Emotional Labour Scale (ELS) specifically for nurses in 2019 [25]. Applying this scale is essential for assessing nurses’ emotional labour and enhancing the quality of care provided, offering valuable insights for nurses and healthcare administrators. The evaluation of this questionnaire demonstrates its alignment with the emotional labour required in nursing, underscoring the importance of translating and validating a comprehensive questionnaire for use in Iran. In Iran, the need to assess the concept of emotional labour among nurses is highlighted by the shortage of nursing staff and the resulting increase in workload. Moreover, religious and cultural beliefs often prompt nurses to neglect self-care and work beyond their capacity. Additionally, having a comprehensive, valid, and reliable questionnaire to measure nurses’ emotional labour could significantly benefit research, education, clinical practice, and management. However, the lack of such a questionnaire, particularly one adapted to the Persian language, presents a significant barrier. Therefore, the present study aims to investigate the reliability and validity of the Persian ELS for nurses in the Iranian healthcare context. Additionally, in light of the stated objective, this study addresses the following questions: What is the validity and reliability of the Persian version of the Emotional Labour Questionnaire for nurses? Furthermore, does the Persian version of the questionnaire exhibit suitable psychometric properties within the Iranian population?

### Methods

This methodological cross-sectional study was conducted between October and December 2024. The study population consisted of nurses working in hospitals in Amol, Iran. Data were collected using a convenience sampling method. The inclusion criteria were: willingness to participate in the study, holding at least a bachelor’s degree in nursing, and having a minimum of six months of work experience. Participants who submitted incomplete questionnaires were excluded from the study. MacCallum et al. suggested that a sample size of at least 200 participants is necessary to conduct psychometric studies [27].

Ultimately, based on the sample size criteria, a total of 434 participants were included in this study. The total sample was randomly divided into two equal groups: the first group (*n* = 217) was used for Exploratory Factor Analysis (EFA), and the second group (*n* = 217) was used for Confirmatory Factor Analysis (CFA). After fully explaining the study objectives, participants were asked to complete the scales. The researcher distributed the scales in person, provided the necessary explanations, and encouraged voluntary participation. Participants were also assured that their participation was completely confidential and that all information would remain anonymous.

### Scale

In 2018, Hong et al. developed and validated the ELS in China. This Scale consists of 16 items across three factors: “Emotional control effort in profession” (7 items), “Patient-Focused Emotional Suppression” (5 items), and “Emotional pretence by norms” (4 items). The scale uses a five-point Likert rating from “1 (not at all)” to “5 (very true).” The total score ranges from 16 to 80, with higher scores indicating more emotional labour [28].

### Scale translation

First, written permission to use the ELS was obtained from the developer of the scale to conduct this research. Then, following Gudmundsson’s [29] translation protocol, the scale was translated from English into Persian. Two translators, fluent in both English and Persian, independently translated. An expert panel, comprising two of the authors of this paper and two professional translators, carefully reviewed and combined the translations to develop the final Persian version of the ELS. Subsequently, the scale was back-translated into English. Finally, the expert panel evaluated and approved the finalized version.

### Validity and reliability

### Face validity

To determine the face validity, it was administered to 10 nurses who met the inclusion criteria. The experts then shared their views on the clarity, content, readability, simplicity, and comprehensibility of the questions.

### Content validity

Both qualitative and quantitative methods were used to assess content validity. For the qualitative assessment, a structured questionnaire was distributed to 10 faculty members from the School of Nursing at Mazandaran University of Medical Sciences. They were asked to provide feedback on grammar, clarity of concepts, and the appropriateness of item phrasing and placement.

For the quantitative assessment, 10 experts, including specialists in psychometrics, nursing education, evaluated each item using an online structured form. The evaluation was based on the Content Validity Ratio (CVR) and Content Validity Index (CVI). According to Lawshe’s method (1975), a CVR value of at least 0.62 was considered acceptable for ten experts, and a CVI value of ≥ 0.70 was used as the minimum threshold for item retention [30].

### Construct validity

To determine the construct validity, an EFA was conducted on the initial dataset (*n* = 217) using Principal Component Analysis (PCA) with varimax rotation. The Kaiser–Meyer–Olkin (KMO) measure of sampling adequacy and Bartlett’s Test of Sphericity were used to evaluate the suitability of the data for factor analysis [31]. Then, only items with eigenvalues greater than 1, Communality above 0.4, and factor loadings higher than 0.3 were retained in the model [32]. In CFA, model fit was assessed by calculating several standard model fit indices, including *χ*², CMIN/DF, CFI, GFI, TLI, NFI, PCFI, PNFI, IFI, and RMSEA [33, 34].

### Convergent and divergent validity

The approach proposed by Fornell and Larcker was used to assess convergent validity. In this process, Average Variance Extracted (AVE) and Composite Reliability (CR) were calculated for each construct. When the AVE of a construct exceeds 0.50, it indicates that the construct explains, on average, more than half of the variance in its indicators, which is considered a criterion for confirming convergent validity. Furthermore, the CR value should exceed 0.7 to confirm construct reliability [35]. Hair et al. suggest that if the CR is sufficient, a marginally lower AVE value may be acceptable without undermining convergent validity [36].

### Reliability

Cronbach’s alpha (α) was calculated to assess internal consistency. CR, McDonald’s Omega (Ω), and Maximum Reliability) MaxR (values were also calculated. For all indices, values greater than 0.7 were considered acceptable thresholds.

### Normal distribution and outliers

In this study, skewness (± 3) and kurtosis (± 7) indices were used to examine the univariate distribution to investigate whether the data were normally distributed. Outliers and missing data were also examined. Multivariate normality was assessed using Mardia’s multivariate skewness coefficient (< 8). Identification of multivariate outliers was performed using the Mahalanobis squared distance statistic (*p* < 0.001) [37, 38]. Normality of the data was confirmed by the Kolmogorov–Smirnov test. All statistical analyses were performed using SPSS and AMOS version 26 software.

## Results

### Demographic characteristics

Of the 434 participants, the mean age was 32.23 (SD = 8.01) years. Among them, 118 (27.2%) were male and 316 (72.8%) were female. A total of 113 participants (52.1%) were married. Regarding educational attainment, the majority held a bachelor’s degree (348 participants, 80.2%).

### Face validity

The evaluation of expert opinions and the target group members indicated that all items had appropriate clarity and relevancy.

### Content validity

The qualitative assessment by 10 nursing faculty members indicated that all items were clear, relevant, and appropriately worded for the target population. Quantitative evaluation showed that all items had acceptable CVR values above 0.62 and CVI values exceeding 0.79, demonstrating acceptable content validity for all items.

### Construct validity

The findings of the KMO index (0.864) and Bartlett’s test (χ² = 1323.343, df = 91, *p* < 0.001) confirmed the suitability of the data for EFA. Results revealed a three-factor structure, consisting of a total of 14 items that explained 61.70% of the total variance. Two items (e.g., “I consciously control my facial expression, attitude, and way of speaking when interacting with patients” and “Although patients make me emotionally uncomfortable, I treat them with positive facial expression and attitude changes instantly”, were excluded due to low factor loadings (< 0.3) and weak communalities (< 0.4), indicating poor psychometric contribution in the Iranian context. Table 1 shows the EFA results.

**Table 1:** EFA results of the Persian version of the ELS

Factor	Items	Factor loading	h^2^	λ	% Variance
	“I try to be kind to patients genuinely from my heart.”	0.730	0.566		
Emotional control effort in the profession	“I try to change my emotions to the positive forms that patients expect.”	0.737	0.643	1.60	11.44
	“I try to adjust my emotions and attitude depending on patients’ emotional change.”	0.677	0.632		
	“I manage my expression and way of speaking with professional attitude to maintain patients’ trust.”	0.752	0.613		
	“I try to understand the different circumstances between doctors and patients.”	0.670	0.584		
	“I try to overcome emotionally challenging situations with a sense of vocation as a nurse.”	0.531	0.541		
	“I suppress my anger when patients’ words and behaviors are unfair.”	0.789	0.710		
Patient-focused emotional suppression	“I tolerate verbally and non-verbally violent behavior from patients, even though I feel scared.”	0.775	0.669	5.70	40.73
	“I tolerate patients expressing negative emotions about medical staff or other departments to me.”	0.783	0.692		
	“I control my mind, thinking that patience is a virtue.”	0.740	0.659		
	“I tolerate unfair treatment to maintain a good work atmosphere on the ward.”	0.625	0.595		
	“I express my emotions to maintain continuous rapport with patients.”	0.504	0.519		
Emotional pretence by norms	“I exaggerate expressions of interest in patients.”	0.793	0.647	1.33	9.51
	“I pretend to feel what I do not actually feel when I deal with patients (e.g., empathy, interest, friendliness, delight, etc.).”	0.710	0.567		

Abbreviations. h^2^: Communalities, λ: Eigenvalues

### The CFA results

**Fig. 1:**
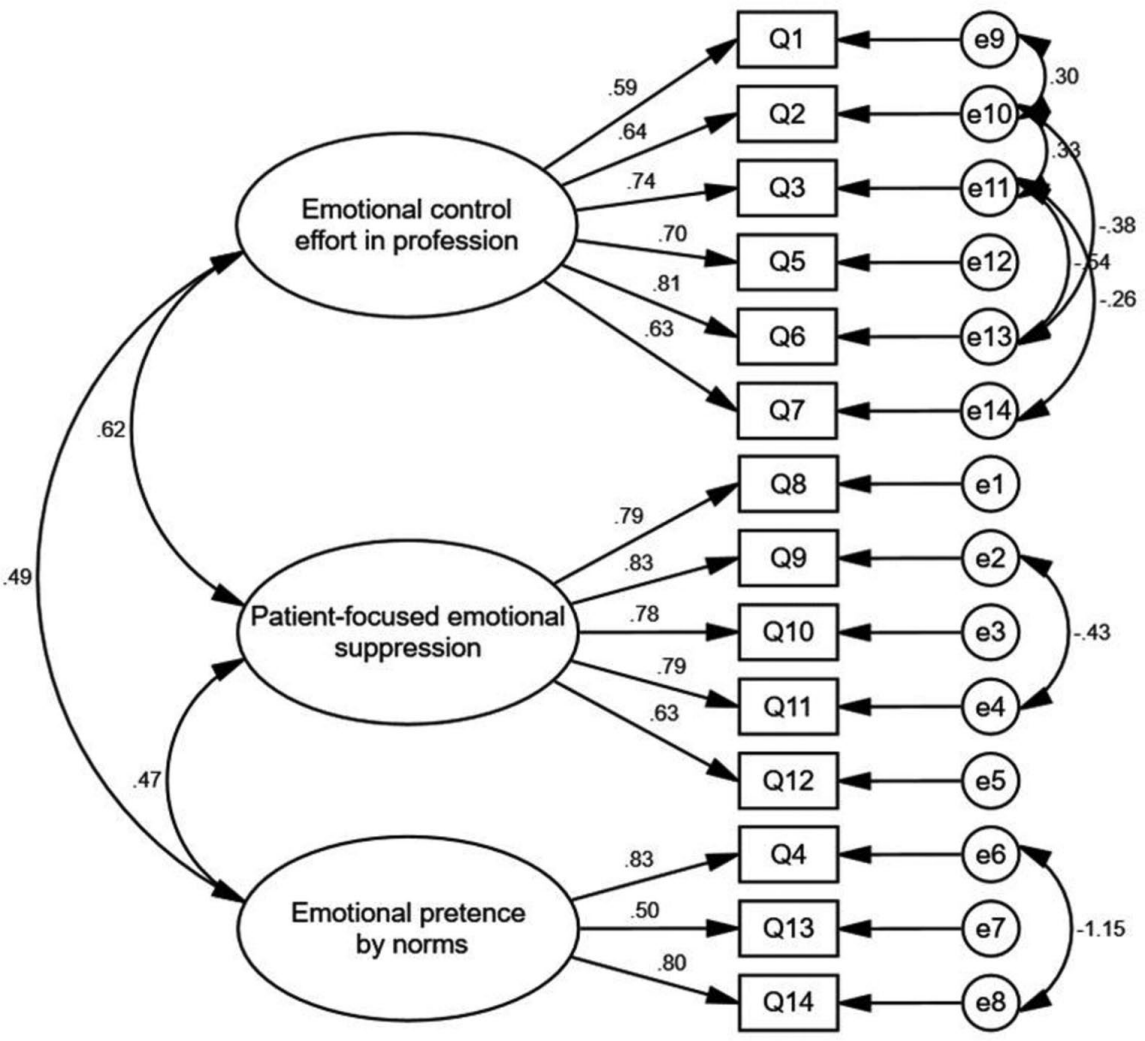
The CFA results

The CFA results demonstrated the goodness of fit of the factor structure by EFA, with the following fit indices: χ² = 131.788, df = 67, χ²/df = 1.967, CFI = 0.94, IFI = 0.95, TLI = 0.93, GFI = 0.92, PCFI = 0.69, PNFI = 0.66, and RMSEA = 0.067. (Acceptable ranges: χ²/DF < 3 is good; TLI, IFI, CFI, GFI > 0.9; PNFI, PCFI > 0.5; RMSEA < 0.08). The three-factor structure was supported by the CFA findings, indicating that the factor solution was stable and consistent across both analyses. Figure 1 shows the CFA results.

### Convergent and divergent validity and reliability

The AVE values for the three factors were 0.474 (Emotional Control Effort in the Profession), 0.590 (Patient-Focused Emotional Suppression), and 0.531 (Emotional Pretence by Norms). The corresponding CR values were 0.842, 0.877, and 0.765, respectively. These values indicate acceptable convergent validity, particularly considering that CR values exceed 0.7 and AVE values are near or above the recommended threshold of 0.50.

Additionally, Cronbach’s alpha, Omega McDonald, and MaxR values for all factors, except the “Emotional pretence by norms” factor, were above 0.7, indicating high internal consistency and acceptable construct reliability. Table 2 shows the results of convergent validity and construct reliability. In Table 3, item–total correlations ranged from 0.371 to 0.677 in the 14-item ELS. None of the items, if deleted, led to a substantial increase in Cronbach’s alpha, supporting the retention of all items.

**Table 2:** Convergent validity and construct reliability results

Factors	α	Ω	CR	MaxR	AVE	MSV
Emotional control effort in the profession	0.831	0.834	0.842	0.857	0.474	0.383
Patient-focused emotional suppression	0.865	0.867	0.877	0.886	0.590	0.383
Emotional pretence by norms	0.643	0.658	0.765	0.816	0.531	0.242

Abbreviations: α: Cronbach’s alpha, Ω: McDonald’s omega

**Table 3:** Item analysis results

Items	Mean	SD	Item–total correlation	Cronbach’s α if the item is deleted
Q1	4.2535	0.808	0.520	0.875
Q2	3.8203	1.009	0.573	0.872
Q3	3.6129	1.039	0.610	0.870
Q4	3.3041	1.174	0.536	0.874
Q5	4.0276	0.917	0.525	0.874
Q6	3.7097	0.929	0.558	0.873
Q7	3.8986	0.896	0.492	0.876
Q8	3.3272	1.092	0.677	0.867
Q9	3.2627	1.190	0.629	0.869
Q10	3.2442	1.182	0.655	0.867
Q11	3.6820	1.038	0.656	0.868
Q12	3.0737	1.230	0.569	0.872
Q13	2.0184	1.239	0.371	0.883
Q14	2.4009	1.221	0.414	0.880

### ELS score

The mean score of emotional labour among the study participants was 47.63 (SD = 9.45). An independent samples *t*-test revealed no statistically significant difference in emotional labour scores between male 46.77) SD = 8.50) and female participants 47.95 (SD = 9.78), *p* > 0.05. Similarly, no significant difference was observed between single 46.27 (SD = 8.50) and married individuals 48.91 (SD = 9.92), or between participants with a bachelor’s degree 47.97 (SD = 9.01) and those with a master’s degree 46.45 (SD = 11.13), *p* > 0.05 for both comparisons. Pearson correlation analysis revealed a statistically significant but weak positive correlation with age (*r* = 0.20, *p* = 0.03), and a marginally significant correlation with work experience (*r* = 0.13, *p* = 0.05), with the emotional labour score.

## Discussion

This study was conducted to assess the reliability and validity of the Persian version of the ELS for nurses. These 14 items collectively explained 61.7% of the total variance. The three factors of the questionnaire are “Patient-focused Emotional Suppression”, “Emotional Control Effort in Profession”, and Emotional Pretence by Norms,” which explain 40.73%, 11.44%, and 9.51% of the total variance, respectively.

In other studies that have psychometrically evaluated this scale in different contexts, the number of items has varied. For instance, in the study by Yao et al., this scale was psychometrically assessed with 16 items among Chinese nurses, with no change in the number of items compared to the original questionnaire [39]. In our study, two items were removed due to inadequate factor loading, which may reflect cultural and religious contextual differences in Iran. As mentioned, in our study, items 15 and 16 with the titles “I consciously control my facial expression, attitude, and way of speaking when interacting with patients” and “Although patients make me emotionally uncomfortable, I treat them with positive facial expression and attitude change instantly” were removed. The nursing context in Iran is shaped by Iranian-Islamic cultural and religious norms, where the open expression of emotions between male nurses and female patients, and vice versa, is often restricted. These sociocultural limitations may justify the removal of item 16. Item 15 focuses on emotional regulation in interactions with patients. Several contextual factors may influence responses to this item, including the moderate to low levels of psychological empowerment among Iranian nurses and the high workload commonly experienced in the nursing profession in Iran. According to Royan et al., psychological empowerment among Iranian nurses is generally moderate and requires further enhancement [40]. Low psychological empowerment weakens nurses’ communication and self-control. A common point between this research and other psychometric studies was the three-factor structure. Other studies investigating this scale have also structured the emotional labour concept into three main factors [28, 39]. Our study found that the 14-item factors collectively predict 61.7% of the total variance of emotional labour, consistent with Yao et al.’s study, which reported that this scale explains 61.28% of the total variance [39].

Reliability of a scale encompasses various aspects, including stability, internal consistency, and equivalence. Internal consistency, or the coherence among the items of a scale, is one of the most important aspects of reliability and indicates that a scale measuring emotional labour should not include items measuring other concepts [41]. In this study, internal consistency was obtained as α Cronbach equal to 0.78. Yao et al., who psychometrically evaluated the same scale in the Chinese context, reported an internal consistency coefficient of 0.817, which is not significantly different from our study. The slight difference between the results of this study and ours is probably due to the removal of two items from our questionnaire [39]. Brotheridge and Lee, who psychometrically evaluated a 15-item emotional labour scale, reported an internal consistency of α Cronbach equal to 0.82 [23]. The content of the items was similar to that in our study. Another study reported an internal consistency coefficient of 0.82 for a questionnaire with similar items to measure emotional labour, which is close to our results [42].

The factors of the ELS encompass items that reflect how nurses control, suppress, or artificially express their emotions in various professional situations. For instance, the factor titled “Emotional Control Effort in Profession” includes items that capture nurses’ attempts to self-regulate their emotional responses during clinical interactions [43]. This factor represents the concept of deep action in emotional labour theory [44]. Deep acting refers to the process by which individuals actively attempt to modify their internal emotional state to genuinely experience the emotions they are expected to display at work. Unlike surface acting, which involves merely faking emotions, deep acting aims to make emotional expressions more authentic and congruent with internal feelings [45]. The concept of emotional labour was first introduced by Arlie Hochschild in 1983, who defined it as the management of emotions to create publicly observable facial and bodily expressions as part of one’s professional role [46]. In this scale, the factors of “patient-focused emotional suppression” and “Emotional Pretence by Norms” refer to the concepts of surface acting in Hochschild’s theory and involves only outwardly displaying emotions without altering true feelings. These factors include items that address the nurse’s exaggerated expression or suppression of emotions in the nurse-patient relationship. In the Emotional labour theory, the concept of surface acting expresses that Employees are expected to evoke or suppress specific emotions to meet organizational or job-related expectations. This often means displaying emotions that are not genuinely felt (e.g., smiling when frustrated) or suppressing emotions that are felt but not appropriate to express in the workplace, and this is exactly what the factors and items of the ELS refer to [47]. Therefore, the factors and items of this scale refer to the items presented in the emotional labour theory under the titles Deep acting and surface acting.

High emotional labour in nurses is associated with negative outcomes such as job burnout and intention to leave the nursing profession [14, 48]. For this reason, the importance of emotional labour and its negative consequences in nurses and nursing care has been emphasized, and studies recommend further attention to this concept and psychometric evaluation of appropriate scales to measure it in nurses across different contexts [14, 49]. Therefore, the psychometric evaluation of this scale can provide accurate insights into the levels of emotional labour among nurses. These data can inform nursing managers in designing and implementing targeted interventions to reduce their negative consequences.

## Conclusions

The present study demonstrated that the Persian version of the ELS, comprising 14 items across three factors, is a valid and reliable instrument for assessing emotional labour among Iranian nurses. The three-factor structure accounted for 61.7% of the total variance, indicating a solid conceptual representation.

The internal consistency of the scale was generally strong, with Cronbach’s alpha values ranging from 0.643 to 0.865 and CR values exceeding 0.76 across all factors. Convergent validity was supported by acceptable average AVE, particularly for the second and third factors, while discriminant validity was confirmed through comparisons with MSV values.

These findings highlight the robust psychometric properties of the Persian ELS in Iran’s cultural and professional nursing context. Much like a well-calibrated diagnostic tool, this scale can sensitively detect variations in nurses’ emotional labour experiences. As such, it offers a practical and evidence-based instrument that can guide nursing managers and policymakers in developing targeted interventions to support emotional well-being, prevent burnout, and ultimately improve the quality of patient care.

### Limitations

This study has several limitations. First, the use of a convenience sampling method may limit the generalizability of the findings to the broader nursing population. Second, the data were collected from nurses working in hospitals in a single geographic area (Amol, Iran), which may reduce the external validity of the results. Third, since emotional labour is a sensitive and psychologically complex subject, some participants may have responded cautiously or with social desirability bias, which could affect the accuracy and honesty of their responses.

Finally, the removal of two items during the cultural adaptation process may limit the conceptual equivalence of the Persian version of the scale compared to the original version. This change could potentially reduce the comparability of results with studies that used the full 16-item version of the scale.

### Implications in nursing

From the standpoint of nursing management, proactively managing emotional labour, burnout, and staff turnover is essential for improving organizational efficiency and ensuring sustainable workforce performance. The validated Persian version of the ELS offers a practical means to assess the emotional labour experienced by nurses systematically. Incorporating the ELS into routine staff assessments, such as during annual evaluations or wellness programs, can help identify individuals at risk of emotional labour and guide the implementation of timely support interventions.

By identifying patterns of emotional labour, nursing managers can develop evidence-based strategies, such as emotional regulation training, peer-support groups, and stress reduction workshops. These interventions can potentially lead to improved job satisfaction, reduced absenteeism, and lower turnover intentions, as shown in previous research.

In nursing education, the ELS can be employed to assess emotional competence among students, helping educators recognize those who may require support in handling emotional stressors. Integrating the scale into training curricula can guide the development of targeted modules to enhance emotional regulation, empathy, and interpersonal communication. Such initiatives may ultimately improve clinical performance, reduce emotional fatigue during clinical placements, and prepare students for the emotional challenges of professional nursing roles.

### Recommendations for future research

Future studies are encouraged to examine the relationship between emotional labour and professional outcomes such as burnout, job satisfaction, and turnover intention. Exploring these associations could provide deeper insight into the impact of emotional labour on nurses’ mental health and performance, and inform the development of comprehensive support systems within healthcare organizations.

## Data Availability

The datasets used and/or analyzed during the current study are available from the corresponding author on reasonable request.
